# A Review of Chondroitin Sulfate’s Preparation, Properties, Functions, and Applications

**DOI:** 10.3390/molecules28207093

**Published:** 2023-10-15

**Authors:** Qingshan Shen, Yujie Guo, Kangyu Wang, Chunhui Zhang, Yanli Ma

**Affiliations:** 1 Zhang Zhongjing College of Chinese Medicine, Nanyang Institute of Technology, Changjiang Road 80, Nanyang 473004, China; 2 Key Laboratory of Agro-Products Processing, Ministry of Agriculture and Rural Affairs, Institute of Food Science and Technology, Chinese Academy of Agricultural Sciences, Beijing 100193, China

**Keywords:** chondroitin sulfate, preparation, property, function and application

## Abstract

Chondroitin sulfate (CS) is a natural macromolecule polysaccharide that is extensively distributed in a wide variety of organisms. CS is of great interest to researchers due to its many in vitro and in vivo functions. CS production derives from a diverse number of sources, including but not limited to extraction from various animals or fish, bio-synthesis, and fermentation, and its purity and homogeneity can vary greatly. The structural diversity of CS with respect to sulfation and saccharide content endows this molecule with distinct complexity, allowing for functional modification. These multiple functions contribute to the application of CS in medicines, biomaterials, and functional foods. In this article, we discuss the preparation of CS from different sources, the structure of various forms of CS, and its binding to other relevant molecules. Moreover, for the creation of this article, the functions and applications of CS were reviewed, with an emphasis on drug discovery, hydrogel formation, delivery systems, and food supplements. We conclude that analyzing some perspectives on structural modifications and preparation methods could potentially influence future applications of CS in medical and biomaterial research.

## 1. Introduction

Chondroitin sulfate (CS) is a typical sulfated glycosaminoglycan (GAG) that has been investigated for many years. The first known report regarding CS, according to the PubMed database, was published in the 1940s. The molecule’s special structural properties, including its highly anionic nature, which is derived from the molecule’s many sulfate or carboxyl groups of CS, allow for a host of various structural types and derivatives to exist, with each of these being responsible for imparting diverse biological functions to CS that influence many pathological processes [1]. For instance, the sulfation patterns of CS, called “sulfation code”, can lead to structural complexity and influence biological functions. Defects in the degree of CS sulfation are often associated with skeletal dysplasia, degenerative diseases, and malignant tumors. The expressions of sulfate groups/sulfation sequences (CS-A and CS-E; CS-C and CS-C) in breast cancer tissue differ. One study indicated that CS-synthesized disaccharides with discrepant sulfation patterns have differential effects on different types of breast cancer cells [2]. Under the effect of a negative charge, CS is allowed to interact with proteins in the extracellular matrix, regulating cellular activities [3]. Moreover, modifications brought about via the interactions of metal ions and peptides, which lead to the formation of a complex, have been of great interest to researchers over the past few years [4,5,6,7,8]. CS is commonly found in animal cartilage and some other connective tissues, including blood vessels, ligaments, skin, and tendons, as well as axon terminals around neuronal cell bodies, the brain, and cells surrounding the extracellular matrix [3]. China has been the largest producer and exporter of CS, with nearly 80% of the world’s CS products coming from China. Most CS products are extracted from animal (e.g., bovine, porcine, and chicken) and marine (e.g., cartilaginous fish, sharks, skate, and bony fish) tissues in factories. The CS end-products produced from these mixed sources may have different characteristics, including purity, biological effects, the presence of contaminants, and clinical efficacy, which may cause quality and safety problems [9]. The present CS extraction methods that involve the use of alkalis or other organic chemicals are counterproductive to eco-friendly production. In addition to animal or marine source extraction, CS can be acquired by adopting bio-synthesis and fermentation methods [10]. Notably, a recent study reported that combining bio-synthesis and fermentation via the engineering of *Pichia pastoris* could serve as the cell factory to prepare type A CS [11], helping to alleviate the shortage of animal extraction sources in the future. Due to its multiple functions and bioactivities, CS is widely applied in medicines, biomaterials, and food supplements. For example, CS has been employed as a slow-acting drug against osteoarthritis [12,13], and it was accepted officially by the World Health Organization/International Liege Against Rheumatism Task Force in 1994. CS is currently being used as a dietary supplement in the United States to treat osteoarthritis, and it is used as a symptomatic slow-acting drug in Europe and some other countries. Some review papers have indicated that CS, together with glucosamine in the clinic, is safe and effective for the treatment of knee osteoarthritis via meta-analysis [14,15]. Additionally, CS is employed in hydrogels, scaffolds, and delivery systems as a biomaterial [16], and it can be used as a functional food for bone care [17], obesity treatment [18], and the regulation of gut microbiota [19]. At present, apart from the clinical data confirming CS’s pain-reducing properties when applied as a drug, other applications of CS, such as its use as a biomaterial or functional food, are mostly based on animal experiments. Here, we systematically summarize the preparation, properties, functions, and applications of CS and propose some ideas for further research.

## 2. Preparation of CS

### 2.1. The Sources of CS

**Figure 1:**
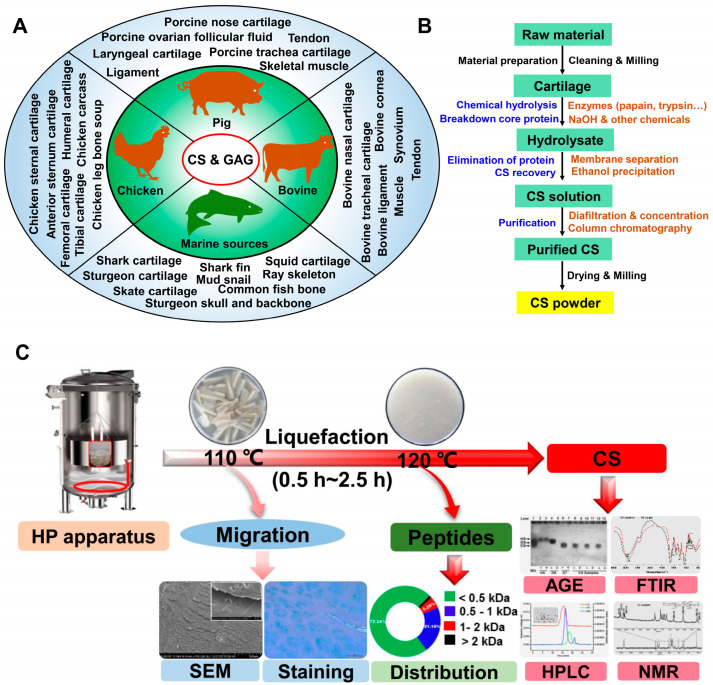
CS sources and extraction methods based on the use of cartilage. (**A**) The main sources of CS and GAG; (**B**) the conventional extraction processes for CS based on the use of cartilage; (**C**) co-production of CS and peptides from chicken sternal cartilage via hot pressure treatment [24] (copyright © 2023, Elsevier). SEM: scanning electron microscopy photograph; AGE: agarose-gel electrophoresis; FTIR: fourier transform-infrared spectroscopy; HPLC: high performance liquid chromatography; NMR: nuclear magnetic resonance.

With the increase in meat consumption, a large number of edible by-products, including animal cartilage, are produced in slaughterhouses. The polysaccharides or proteins in animal cartilage can be recycled for high-value product ingredients in industry. The majority of CS is obtained from animal sources (mainly from animal cartilage and connective tissue) and marine sources via various extraction methods (Figure 1A).

*Chicken source*. Due to the lack of religious restrictions regarding its consumption, chicken meat is one of the most popular meats worldwide. A USDA report from 2019 (https://www.fas.usda.gov/data/china-poultry-and-products-annual-3, accessed on 24 July 2023) suggests that China’s annual chicken meat production is 13.8 million metric tons, which means that considerable by-products (e.g., bone, cartilage, and connective tissue) are produced. In particular, chicken sternal cartilage is usually made into snack foods (cartilage and chicken meat) in China for its rich nutrients (e.g., collagen and polysaccharides). Interestingly, it has been reported that CS and CS peptides can be extracted from chicken keel cartilage [20,21,22,23,24]. Other parts, including the anterior sternum cartilage, proximal humeral cartilage, distal humeral cartilage, proximal femoral cartilage, distal femoral cartilage, proximal tibial cartilage, and distal tibial cartilage of broiler chicken, can be employed for CS peptide extraction via tissue autolysis with 0.5 mol/L sodium acetate buffer [25]. Additionally, the chicken leg bone (i.e., the ends of the leg bone) contains polysaccharides (mainly CS), and this has been verified via alcian blue staining, and bone soup can be employed for CS isolation [26]. Moreover, broiler chicken by-products from mechanical deboning, such as crushed bones, cartilage, skin, adipose tissue, and muscle, can also be used to isolate CS [27]. As indicated by the results of one specific work in the literature, CS can be extracted from this by-product via proteolysis. Besides the cartilage, the uronic acid content of GAGs can be determined in the sternum bone, keel cartilage perichondrium, coracoid bone, skin, adipose tissue, and hip muscles, with different concentrations ranging from 0.7 to 11 μg/mg. The sternum bone especially has the highest concentration. After confirmation, CS is the major GAG in the above-described samples. Similar research suggests that sulfated glycosaminoglycan is capable of being isolated from the wing bones, leg bones, front bones, and hind bones of chicken carcasses, and 1.9 g CS can be acquired from a 1.66 kg whole broiler chicken carcass [28]. Similarly, the cartilage or cartilage-like tissues of other poultry (e.g., duck, goose, and birds) by-products can theoretically be employed to prepare CS, although research on this and related topics has been reported in the literature. This may be due to their limitations in quantity compared to chicken by-products.

*Bovine source*. Bovine by-products are another primary source of CS. Bovine nasal cartilage, tracheal cartilage, and corneas are all common raw materials used to prepare CS. A recent study indicated that CS could be isolated from bovine nasal cartilage after being subjected to papain and trypsin treatment, and the smallest molecular weight CS fraction exhibited a stronger anti-oxidant activity [29]. Another study reported the extraction of CS and CS proteoglycans from bovine tracheal cartilage; in this study, the CS chains were heterogeneous, with different levels of chondroitin 4- and 6-sulfates [30,31]. In addition to cartilage, other non-cartilaginous tissues, such as corneas and other connective tissues, can be used for CS isolation. Corneas from bovine eyes can be employed for CS proteoglycan extraction, and this corneal CS proteoglycan can be applied in investigations regarding the structural interactions involved in the adherence of *Plasmodium falciparum*-infected red blood cells [32]. In terms of connective tissues other than cartilage, decorin and biglycan have been reported as two predominant proteoglycans in bovine tendons and ligaments [33]. In addition, CS and other sulfated GAGs can be extracted from the connective tissues of bovine (e.g., the tendon, ligament, muscle, and synovium) [34].

*Porcine and other animal sources*. Porcine by-products are another source of CS. Specifically, porcine nose cartilage, laryngeal, and trachea cartilage have been studied for their use in the preparation of CS [35,36,37]. Moreover, porcine ovarian follicular fluid can be used to isolate proteoglycans, including CS and dermatan sulfate [38]. In porcine tendons and ligaments, the majority of GAGs are hyaluronan, followed by dermatan sulfate and small amounts of CS [39]. In porcine skeletal muscle, sulfated GAG can also be isolated [40]. Other animal sources, including sheep nasal cartilage [41] and growing antlers [42], have also been reported on for their use in CS extraction. CS has been chemically studied in relation to pilose antlers [43,44], and GAGs can be isolated from the different parts of growing antlers of wapiti; it has been confirmed that, on average, the total uronic acid of GAG is 88% in each section of antler.

*Boney fish and other marine sources*. Although the majority of commercial-grade CS originates from animal sources (e.g., cartilage or non-cartilaginous tissues of chicken, bovine, porcine, sheep, and other terrestrial animals), some issues regarding these animal sources, including religious restrictions and safety (e.g., mad cow disease, H7N9 avian influenza, foot and mouth disease, hog cholera, and other food chain crises), should be considered. The above-mentioned problems can be avoided by selecting bony fish and other marine by-products as CS sources. CS from marine sources (shark, sturgeon, and skate) has been shown to exhibit better activity and quality. It has been reported that 132 million tons of marine organisms, including aquaculture (mainly fish, mollusks, and crustaceans), are captured yearly, and over 35% of this total weight comprise by-products or waste [45]. The considerable by-products of cartilaginous materials or bones from fish (e.g., shark, salmon, ray, and common fish) and other marine organisms, such as sea sponges, sea cucumbers, squids, mollusks, and some invertebrates, are good sources for obtaining GAGs [46,47]. For these aquatic organisms, shark cartilage [48,49,50], skate cartilage [51,52], squid cartilage [53,54,55], sturgeon cartilage, the skull and backbone [56,57,58], and mud snails [59] have been investigated to obtain CS via various extraction methods. Although shark fins are a source of high-quality CS, the non-rational exploitation of sharks and other ecological aspects have led many to refrain from using them to extract CS; despite being another attractive source of CS, it is also considered bad practice to use sting ray skeletons to extract CS [45,60,61]. Interestingly, Maccari et al. [62] reported that CS could also be isolated and purified from the bones of common fishes such as monkfish, cod, spiny dogfish, salmon, and tuna, and the contents of CS (in terms of uronic acid concentration, as determined via carbazole assay) range from 0.011% for cod up to 0.34% for monkfish with different structural disaccharides.

### 2.2. CS Extraction from Cartilage

Methods regarding the isolation of CS from cartilage have been investigated for years. In general, the various extraction processes can be condensed into four key steps [45]: the chemical hydrolysis of cartilage, the breakdown of the proteoglycan core, the elimination of proteins and CS recovery, and the purification of CS. Remarkably, the first two steps usually require alkali hydrolysis with high concentrations of NaOH, urea, or guanidine HCl. Subsequently, it combines with other chemicals to selectively precipitate GAG and separate proteins. Finally, purification is performed via gel filtration or ion exchange and size-exclusion chromatography. A flow chart showing the steps required for CS isolation from cartilage is shown in Figure 1B. However, this traditional isolation method is not environmentally friendly and economical, especially when high concentrations of alkalis are used. Recently, due to sustainability concerns, many efforts have been made to reduce NaOH use or replace it. For instance, cartilage digested by different enzymes in a buffer solution (without NaOH) can be used to extract CS [51,63,64]. Proteins (generally those containing type II collagen) are considered the main components of dried cartilage. Accordingly, the co-production of CS and proteins (or peptides) should be considered in practical large-scale production in factories. Although high temperatures may affect the molecular weight of CS or its structure, a previous research study indicated that CS (MW > 35 kDa) could be acquired from bone soup subjected to 120 °C for 2 h [26], and no apparent structural difference was observed compared with the standard. Based on this result, the thermal liquefaction of chicken sternal cartilage was proposed to obtain CS and peptides [24]. The cartilage was liquefied after hot pressure treatment at 120 °C for 1.5 h. After enzymatic hydrolysis with papain and trypsin, CS and peptides were obtained via membrane separation (Figure 1C). These extraction processes hardly contain chemicals aside from enzymes, and they have the potential to be used in factories to facilitate large-scale production.

Based on the conventional CS extraction method and the industrially large-scale production of CS from cartilage, two points should be considered with respect to practical production. The first is the pretreatment of cartilage prior to enzyme hydrolysis. Here, thermal liquefaction is employed instead of simple mechanical treatments such as cutting pieces or milling for the cartilage. In one study, the protein content exceeded 70% in cartilage, and the yield of collagen type II from sternal chicken cartilage exceeded 15% [65]. Type II collagen is a macromolecule that covers three identical α1 polypeptide chains with intact triple helix structures, and the denaturation temperature of type II collagen is only 37–38 °C [66], indicating that high temperatures can influence the structure of collagen. In fact, subjecting cartilage to 120 °C will increase the Brix of the solution by 5%. As a result, the polysaccharides (mainly CS) migrate into the solution due to the cartilage structure change induced by the thermal treatment [24]. Moreover, after thermal treatment, the cartilage will be easier to hydrolyze via enzyme hydrolysis.

Another point is CS recovery. Liquefied cartilage that has been subjected to enzymatic hydrolysis can be used to recover CS. At this stage, two options can be chosen. One is ethanol precipitation, in which purer CS can be obtained after repeating this operation. However, in large-scale production, plenty of ethanol will be consumed, which results in the reagent residual problem and difficulties in peptide recovery. Moreover, using ethanol during recovery will increase costs. An alternative option is membrane separation. Based on differences in molecular weight, different-sized membranes (e.g., ultrafiltration/diafiltration and microfiltration/ultrafiltration/diafiltration) can be combined to isolate crude CS and peptides. Subsequently, pure CS will be obtained after purification via column chromatography. This combined membrane separation technology seems more suitable for continuous production in factories, through which CS and peptides can be co-produced without considering ethanol recovery.

### 2.3. Enzymatic and Chemical Synthesis and Fermentation for CS

Despite the significant efforts that have been made to improve the quality of CS derived from animal or marine by-products in the industry, health, safety, and ecological problems still need to be considered. Strategies including bio-syntheses such as enzymatic and chemical synthesis and fermentation for the production of CS or CS-like products have been proposed to find better solutions.

**Figure 2:**
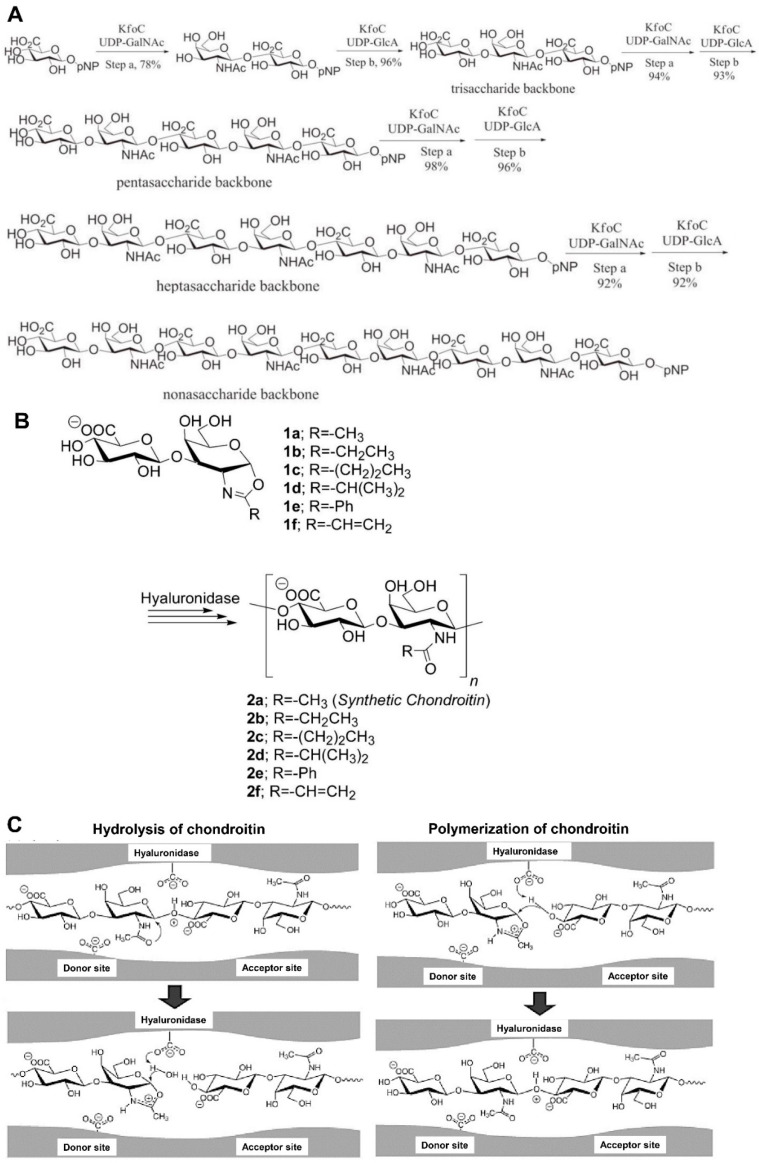
Enzymatic synthesis of CS oligosaccharides. (**A**) Synthesis of the chondroitin backbones by GTases KfoC [68] (copyright © 2023, Wiley-VCH Verlag GmbH & Co. KGaA, Weinheim). (**B**) Enzymatic polymerization of synthetic chondroitin and its derivatives. (**C**) Postulated reaction mechanisms of HAase catalysis for the hydrolysis of chondroitin and for the polymerization of monomer **1a**. [71] (copyright © 2023, American Chemical Society).

*Enzymatic synthesis*. Enzymatic polymerization is recognized as an alternative method for the synthesis of natural or unnatural polymers, including polysaccharides. Two types of CS synthases, including glycosyltransferases (GTases) and hyaluronidase (HAase), have largely been employed for the preparation of homogenous CS [67]. GTases can synthesize CS chain backbones in vitro, which can be achieved by adding the monosaccharide units from uridine diphosphate (UDP)-sugar donors into an acceptor or primer sugar. Li et al. and Sugiura et al. [68,69] reported that GTases KfoC from the *E. coli* K4 strain can synthesize CS oligosaccharide backbones by transferring UDP-GalNAc and UDP-GlcA to the acceptor; this involves the specific steps shown in Figure 2A.

The glycoside hydrolase has been employed in single-step polysaccharide synthesis for enzymatic polymerization. HAase has been reported to catalyze CS synthesis in vitro [70,71]. Although HAase can serve as a hydrolysis enzyme for chondroitin, it can also catalyze the formation of repeated glycosidic bonds in vitro and not just in the catabolic direction. *N*-Acetylchondrosine (GlcAβ (1→3) GalNAc) oxazoline (**1a**) and its derivatives (**1b**–**1f**) are designed and synthesized as transition state analog substrate monomers for catalysis by HAase. 2-methyl (**1a**), 2-ethyl (**1b**), and 2-vinyl (**1f**) oxazoline derivatives are polymerized by HAase via ring-opening polyaddition with the total control of regioselectivity and stereochemistry. As a result, the synthetic chondroitin (natural type; *N*-acetyl, **2a**) and the derivatives (unnatural type) with *N*-propionyl (**2b**) and *N*-acryloyl (**2f**) functional groups are derived at the C2 position of the galactosamine unit. 2-*n*-propyl (**1c**) and 2-isopropyl (**1d**) oxazoline derivatives are polymerized to produce **2c** and **2d**. The 2-phenyl oxazoline derivative (**1e**) cannot afford any enzyme-catalyzed products (Figure 2B). Chondroitin and CS were reported to be hydrolyzed at (1→4)-β-*N*-acetyl-*D*-galactosaminide linkage via HAase catalysis [72]. However, Kobayashi et al. [71] reported a facile and efficient method of synthetic chondroitin (natural type) via enzymatic ring-opening polyaddition, which also involved HAase catalysis. HAase can act as a bifunctional enzyme in hydrolysis and catalysis. For hydrolysis, after the chondroitin substrate is subjected to the HAase, the protonation of the oxygen atom in the β (1→4) glycosidic linkage occurs. Subsequently, the carbonyl oxygen atom from GalNAc will attack its anomeric carbon atom on the α-side, which cleaves the glycosidic bond and produces a high-energy oxazolinium ion species. The oxazolinium anomeric carbon can be attacked by water nucleophilically to open the oxazolinium ring, ultimately resulting in chondroitin hydrolysis [71,73] (Figure 2C). Regarding the polymerization mechanism, especially for monomer **1a**, it is easily recognized by the HAase and activated by protonation at the donor site because of its protonated monomer structure, which is the same as the oxazolinium transition state. Here, monomer **1a** can be recognized as a transition state analog substrate in an activated form, the structure of which can be recognized and activated by the enzyme for the subsequent reactions. The 4-hydroxyl group of GlcA in **1a**, or the nonreducing end of the growing chain placed in the acceptor site, regioselectively adds to the anomeric carbon of the oxazolinium ion of **1a** from the β-side, forming a β (1→4) glycosidic linkage between GalNAc and GlcA [71] (Figure 2C). This regio- and stereoselective glycosylation repetition and ring-opening polyaddition of **1a** is catalyzed by the HAase synthesis of chondroitin.

**Figure 3:**
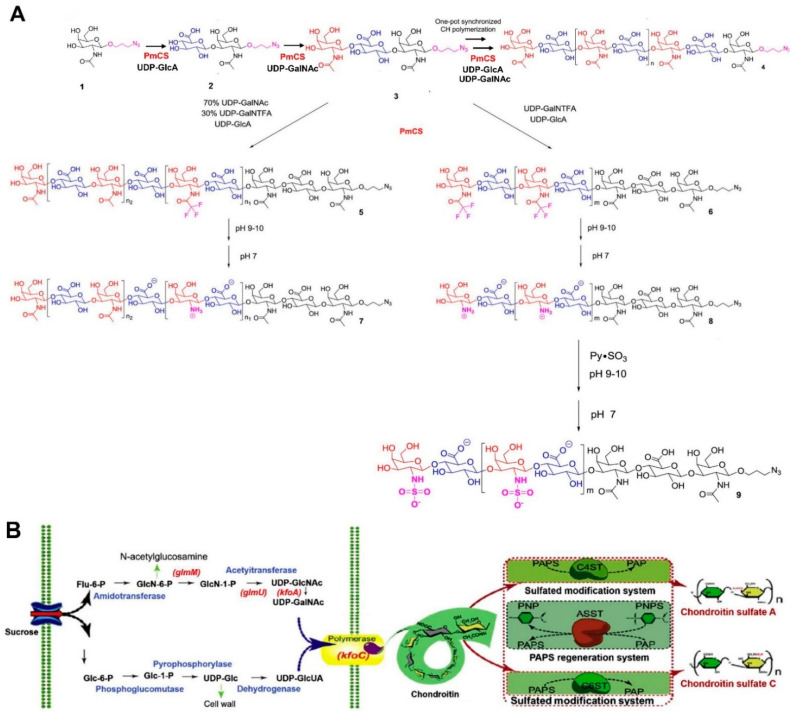
Chemoenzymatic synthesis of CS. (**A**) The common strategy for the enzymatic synthesis of homogeneous chondroitin polymers (**1**–**4**); chemoenzymatic synthetic method for chondroitin derivatives (**3**–**8**); chemical synthesis of *N*-sulfonated CH polymers (**8**–**9**) [82] (copyright © 2023, Elsevier). (**B**) Two-step biological strategy of biosynthesis for producing CS in vivo and in vitro [83] (copyright © 2023, Wiley Periodicals Inc).

*Chemical synthesis*. According to Ji et al.’s classification [67], the chemical synthesis of CS or CS oligosaccharides mainly includes semisynthesis and total synthesis. Semisynthesis begins with a purified CS oligosaccharide acceptor, and total synthesis is based on a linear type of assembly generally derived from monosaccharide building blocks [74]. Recently, fucosylated CS or its oligosaccharides were reported to be synthesized via chemical synthesis [75,76,77]. CS and its subtypes can also be synthesized using semisynthesis or total synthesis methods [78,79,80,81]. Interestingly, except for the pure chemical or enzymatic processes, the combination of these two methods, chemoenzymatic synthesis, has received a great deal of attention recently. Wang et al. [82] employed the chemoenzymatic method to synthesize chondroitin polymers and chondroitin derivatives. In this work, the minimal acceptor was employed, and the polymerization of the homogeneous chondroitin chain was catalyzed by *Pasteurella multocida* (*P. multocida*) chondroitin synthase (*PmCS*) (Figure 3A). The biosynthesis and chemical synthesis methods mentioned above were all achieved in vitro. Zhou et al. developed a two-step method to synthesize CS with commercial sucrose in vivo and in vitro [83]. The chondroitin biosynthesis pathway was investigated, and optimization was carried out to obtain the chondroitin via fed-batch fermentation. Based on the fermentation products, an efficient 3′-phosphoadenosine-5′-phosphosulfate (PAPS) regeneration system and specific sulfation transformation systems, together with the purified aryl sulfotransferase IV, chondroitin 4-sulfotransferase (C4ST), and chondroitin 6-sulfotransferase (C6ST), were established to transform chondroitin into CS (Figure 3B). This method can be an effective alternative biosynthesis method for obtaining CS using inexpensive carbon sources.

**Figure 4:**
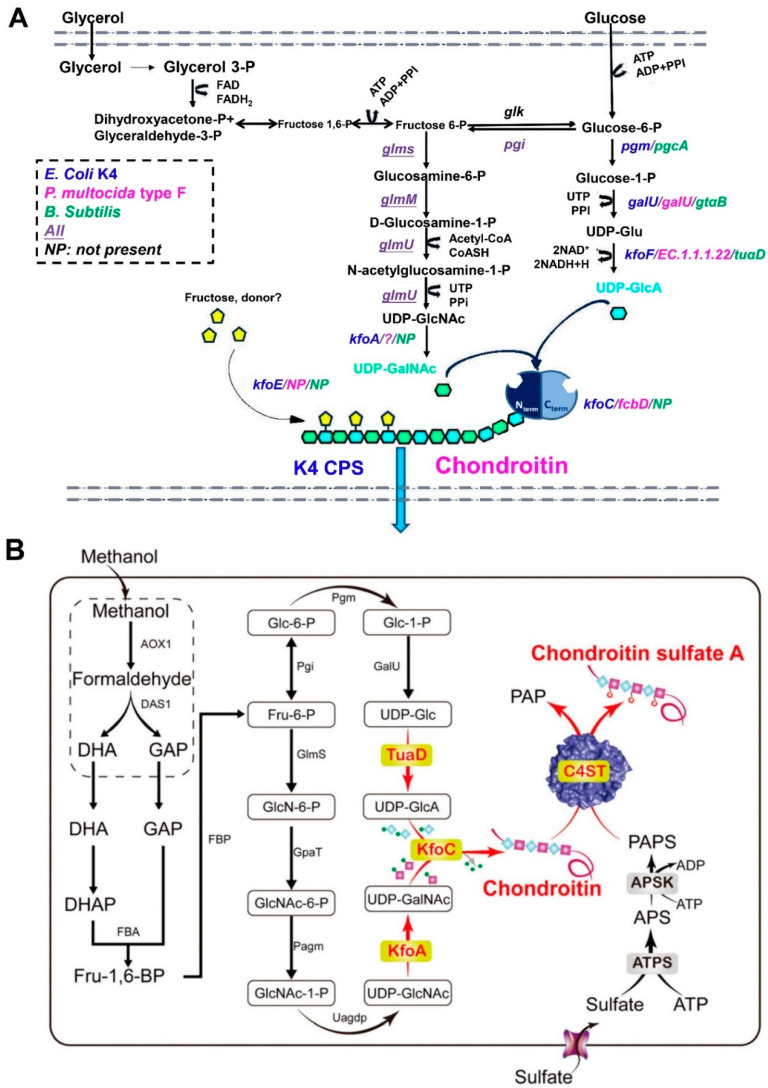
(**A**) Metabolic pathways for the synthesis of fructosylated and defructosylated chondroitin in *E. coli* K4, *P. multocida* type F, and non-native producer *B. subtilis* [91] (copyright © 2023, Portland Press). (**B**) Schematic diagram of chondroitin sulfate A synthesis in engineered *P. pastoris* [11] (copyright © 2023, Royal Society of Chemistry).

*Fermentation for CS*. In addition to the above-mentioned methods, microorganic fermentation can be utilized to obtain CS or CS-like polymers. The most extensively used microbiota are *Escherichia coli* (*E. coli*), *P. multocida*, and *Bacillus subtilis* (*B. subtilis*). Rodriguez et al. [84] initially reported and characterized the K4-specific capsular polysaccharide (CPS) from *E. coli* O5:K4: H. This type of CPS was verified as a fructose-containing polysaccharide with a chondroitin backbone. Subsequently, Manzoni et al. [85] prepared the K4 extracellular polysaccharide from a strain of *E. coli*, resulting in a 200 mg/L yield. Subsequently, numerous researchers have devoted their efforts to improving the yield of CPS from the fermentation products of *E. coli* by modifying fermentation strategies with small-scale batch and fed-batch techniques. For example, Schiraldi et al. [86] developed a simple and economical fermentation strategy using glycerol as the primary carbon source and soy peptone as a complex nitrogen source. The effectiveness of this strategy was aptly demonstrated in successive small-scale batch and fed-batch experiments, with maximum cell densities of up to 56 g_cww_/L and a titer of CPS equal to 1.4 g/L in the fed-batch mode. This was a seven-fold improvement compared to the yield noted in Manzoni et al.’s report. Following these results, the fermentation strategy was further investigated by changing the aeration conditions and the microfiltration bioreactor [87]. The inhibitory effect of acetate on bacterial cell growth and K4 CPS production was analyzed under shake flask conditions, and the feeding profiles were optimized. As indicated by the previous results, the high polysaccharide concentration reached 4.73 g/L, and the increase in K4 CPS titer, compared with the fed-batch results, increased 3.3-fold. Instead of *E. coli*, *P. multocida* and *B. subtilis* were utilized to produce CS or CS-like polymers by the authors [10]. Among the five *P. multocida* capsular groups (A, B, D, E, and F), type D and type F have been used for the preparation of chondroitin or chondroitin-like polymers [88]. In contrast, the pathogenicity of *P. multocida*, such as the major fowl cholera pathogen from Type A, a swine pathogen from Type D, and a minor fowl cholera pathogen from Type F, impeded and reduced its interest in fermentation to a certain extent [86]. Additionally, a patent described by Liu and collaborators [89] screened a new strain of *B. subtilis* BN to produce CS from the fermentation products, and the yield of CS was increased to 4.2 g/L after optimizing the fermentation conditions. Jin et al. [90] obtained chondroitin from recombinant *B. subtilis* fermentation, and the production of chondroitin reached 5.22 g/L. The strains mentioned above, including the pathogenic bacteria of *E. coli* or *P. multocida* and the safer bacteria of *B. subtilis*, can naturally produce the CPS. This capsular polysaccharide was identified as chondroitin, which can be used as a raw material for CS synthesis by adding sulfation groups with chemical or enzymatic methods. In fact, Cimini and collaborators [91] highlighted that these strains have the gene arsenal needed to (i) synthesize building blocks such as UDP-GalNAc and UDP-GlcA, (ii) assemble them, and (iii) transport the CPS out of the cell wall (Figure 4A).

In addition to optimizing fermentation conditions to obtain CS or chondroitin, engineering strain strategies have also been investigated. The combined designed method of engineered microorganisms and fermentation processes with semi-synthetic or enzyme-based approaches has attracted researchers’ attention. For instance, Jin and collaborators [11] recently developed a route for the de novo biosynthesis of CS, starting from methanol by introducing *kfoC* and *kfoA* genes from *E. coli* K4 and the *tuaD* gene from *B. subtilis* into *Pichia pastoris* as an engineered cell factory, constructing the chondroitin synthesis pathway; the final product of CS-A was obtained by integrating sulfonation in the engineered strain (Figure 4B). This cell factory, designed for chondroitin production, is involved in various aspects of strain/pathway engineering, which involves (i) the identification of bottlenecks in polymer assembly and building block provision, (ii) the investigation of regulatory constraints, and (iii) the implementation of the metabolic networks of novel hosts [91]. The genes controlling CPS biosynthesis and transport in *E. coli* K4 include three regions. Specifically, regions 1 and 3, which are found in all group II *E. coli* strains, are responsible for CPS exportation and assembly on the cell surface [92,93]. Region 2 is serotype-specific. It comprises an IS2 insertion sequence and seven genes that are involved in activated precursor biosynthesis (*kfoA*, *kfoF*), polymer assembly (*kfoC*), fructosylation (*kfoE*) [94,95,96], and other unknown genes of (*kfoB*, *kfoD*, and *kfoG*). An overview of the results reported in the literature regarding the use of wild strains and engineered bacteria in fermentation processes is provided in Table 1.

### 2.4. Summary of CS Preparation Methods

As mentioned above, CS and CS-like polymers can be obtained from tissues (main cartilage) of terrestrial or marine animals, via enzymatic or chemical synthesis, and from microbial fermentation. However, each method has its unique characteristics. The advantages and disadvantages of these methods should be considered in practical production. Regarding the raw materials derived from terrestrial or marine species, cartilage (from chicken, bovine, and porcine, or marine sources such as cartilaginous fish, sharks, skate, and bony fish) can be acquired easily due to the increasing production and consumption of animal meat, which can provide sufficient raw materials to prepare commercial-grade CS on a large scale in factories. However, many problems can arise from the use of this type of raw material. Safety and contamination are of critical significance. Animal-sourced CS products may be contaminated easily with several inflammatory agents or pathogens, resulting in anaphylaxis or cross-infection with zoonotic diseases, including hog cholera, foot and mouth disease, and cow diseases [112]. Additionally, these CS products usually exhibit heterogeneity because of the source of cartilage from the terricolous (products mainly contain CS-A) or marine species (products mainly contain CS-C). CS has a complex structure that is strongly correlated with animal tissue, organs, species, and age [9,113,114]. Moreover, other GAGs, such as dermatan sulfate and heparin, exist in these tissues, impacting the homogeneity and purity of the final CS product, limiting biological effects, and restricting their clinical application in medicine. Additionally, restrictions on use related to religious concerns may arise. Lastly, extracting CS from animal tissues often includes high temperatures, strong acids, and alkalis or other chemical treatments, which may trigger concerns regarding eco-friendliness. In summary, the main general characteristics and properties of animal-derived commercial-grade CS encompass safety and quality, as noted by Volpi [9], and include the following aspects: (1) variability and a generally undefined and heterogeneous source of extraction; (2) the possible cross-contamination between sources of different origin; (3) the possible presence of bacteria, viruses, or prions; (4) a high content of proteins that are not characterized (up to 5–10%), and some of these proteins may have allergenic potential able to develop immune reactions; (5) a variable content of immunogenic keratan sulfate and other natural biopolymers and variable purity; (6) a heterogeneous structure, physicochemical properties, and a variable molecular mass, polydispersity, and charge density; (7) the extraction process is generally not controlled, causing possible modifications to the CS structure, such as desulfation or depolymerization; (8) possible intentional adulteration by artificial (macro) molecules; (9) possible batch-to-batch variability; and (10) no evaluation of any biological activity.

With this in mind, a question is raised about how to evaluate the quality, structural characteristics, and other specific parameters of CS to ensure its safety and quality. To the best of our knowledge, except for the common methods (e.g., infrared spectroscopy, specific optical rotation, and intrinsic viscosity), modern methods (e.g., size-exclusion chromatography coupled with different detectors [115], agarose gel electrophoresis, cellulose acetate electrophoresis, high-performance size-exclusion chromatography, and enzymatic HPLC) have been recommended to determine the quality, quantity, chemical properties, and structure of CS [9]. High-quality CS obtained from bovine cartilage manufactured by Bioiberica and CS sodium of marine origin or shark cartilage is commercially available as a reference standard. The safety of the raw materials used is significant for high-quality CS. Of course, through using non-animal sources (e.g., enzymatic or chemical synthesis and fermentation by microbes) of CS, some of the problems mentioned above can be avoided. For example, CS oligosaccharides derived from enzymatic or chemical synthesis have a homogeneous structure [70,74,116,117]; there is no need to consider the religious and pathogenic pollution problems. However, a great challenge for the enzymatic or chemical synthesis of CS is large-scale production due to the complicated synthesis routes and low product yield, the latter of which is essential to meet the demands of the CS market. However, fermentation with an engineering microbe seems to be an effective solution to increase yield and improve the production scale. Most of the fermentation product is the K4 CPS or chondroitin with the furanose residue of fructose instead of a perfect CS structure. Hence, the final products need the follow-up steps of chemical sulfation or fructose monomer hydrolysis. In addition, some microbes, such as *E. coli* and *P. multocida*, are pathogenic, which could compromise product quality. Interestingly, recently, the CS-producing cell factory that combines enzymatic or chemical synthesis and fermentation methods [11,83,90,118] was investigated. With this method, pure CS can be obtained from the fermentation product. This method should be considered as a potential strategy for preparing non-animal CS.

## 3. Properties of Chondroitin Sulfate

### 3.1. Structural Properties

**Figure 5:**
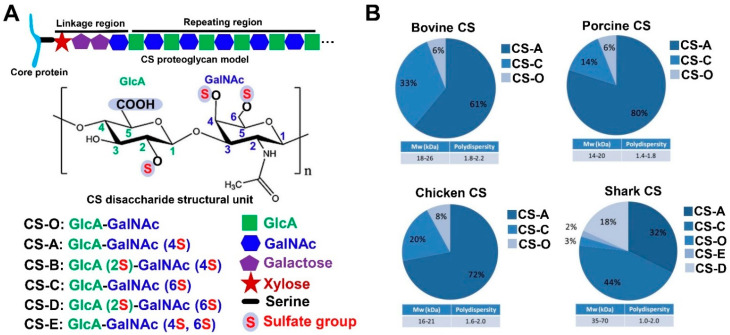
CS structure. (**A**) The structure of the common CS type. S: sulfate group; F: fucosylated group. (**B**) The content and molecular weight of different CS types from bovine, porcine, chicken, and shark sources [91] (copyright © 2023, Portland Press).

CS is formed by a hundred or more repeating disaccharide units [(–4GlcAβ1–3GlcNAcβ1–)_n_], which are the *N*-acetyl galactosamine (GalNAc) residues, substituted to varying degrees with sulfate linked to the 4- or 6-hydroxyl positions, alternating in glycosidic linkages with glucuronic acid (GlcA) substituted with sulfate at the 2- and (more rarely) 3-hydroxyl positions. CS, a typical sulfated glycosaminoglycan, exists on cell surfaces and in extra/pericellular matrices in a proteoglycan form, where the CS chain is covalently attached to a panel of core proteins (Figure 5A). In vivo, CS is synthesized and assembled in the endoplasmic reticulum/Golgi compartments. A tetrasaccharide structure of glucuronic acid-galactose-galactose-xylose is covalently linked to the specific serine residues embedded in the core protein of the CS chain [1,119,120]. Research suggests that the difference in sulfation is attributed to the correct juxtaposition of the related sulfotransferases and acceptor chondroitin domain in a subcompartment of the Golgi network [120,121], mainly involving the chondroitin 6-sulfotransferase, chondroitin 4-sulfotransferase, and uronyl 2-sulfotransferase in adult mammals [122,123]. Yamada et al. [124] reported that chondroitin 4-sulfotransferase-1 and chondroitin 6-sulfotransferase-1 could specifically transfer sulfation from adenosine 3’-phosphate 5’-phosphosulfate to positions 4 and 6 of the GalNAc residues, respectively. CS is classified into ten common types based on the different sulfated locations and degrees, which include CS-O, CS-A, CS-B, CS-C, CS-D, and CS-E, as shown in Figure 5A, and the other four are CS-F (fucosylated carbon 3 of GlcA and sulfated carbon 4 of GalNAc), CS-M (sulfated carbon 3 of GlcA and sulfated carbon 4, 6 of GalNAc), CS-K (sulfated carbon 3 of GlcA and sulfated carbon 4 of GalNAc), and CS-L (sulfated carbon 3 of GlcA and sulfated carbon 6 of GalNAc; sulfated carbon 2, 3 of GlcA and sulfated carbon 4, 6 of GalNAc). The most common CS types are CS-A and CS-C, which, as a drug or commercial product, can be extracted from terrestrial animal and marine fish cartilage such as bovine, pig, chicken, and shark cartilages (see part 3 below), and the composition and CS content of these extracts are varied among these sources (Figure 5B) [91]. Aside from the aforementioned two most common types, 2% and 18% of CS-E and CS-D have been observed in shark CS.

As a polysaccharide, the initial size of CS depends on the degree of polymerization. The overall size of the entire proteoglycan ranges from as small as 80 kDa to as large as 3500 kDa, which includes the core proteins size ranging from as small as 10 kDa to as large as 500 kDa [120]. The molecular weight of the CS product, especially for the extracted CS, can be impacted by various factors, including the CS source and the extraction process (temperature or chemical treatments). The molecular weight of the known CS from the common animal or shark ranges from 14 to 70 kDa, and the polydispersity ranges from 1.0 to 2.0 (Figure 5B). The measuring methods probably lead to molecular weight differences. For example, one study reported the molecular weight of uniform fucosylated CS was to be 27 kDa [125] using TSK gel chromatography method, while a pronouncedly higher value than that (76.4 kDa, 98.1 kDa) was reported in another study (this time measured via high-performance gel permeation chromatography) [126,127]. Therefore, it is necessary to report the molecular weight of CS, along with the determined conditions and methods used. Another physicochemical property of CS is its high viscosity in water solutions (100 mg/mL) with a clear or slightly hazy or faintly yellow compound due to the source. Regarding identification, CS, together with standard GAGs, can be identified via agarose gel electrophoresis [62], and CS type determination can be performed via SAX-HPLC after sample enzymolysis [128]. Furthermore, Fourier-transform infrared spectroscopy and ^1^H and ^13^C nuclear magnetic resonance spectroscopy can also be used to confirm the essential structure of CS, whereas it is necessary to use mass spectrometry coupled with nuclear magnetic resonance or several other methods to determine the fine structure of CS [129,130].

### 3.2. The Complex Properties of CS

CS is a kind of negatively charged polysaccharide, and its structural features suggest that it has many free carboxyl and sulfate groups throughout its chain. This means that CS could interact with metal ions and some other substrates with a positive charge.

**Figure 6:**
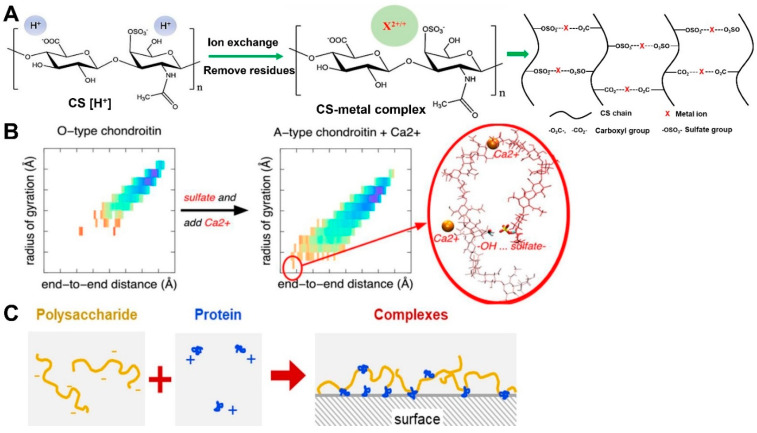
CS complex properties. (**A**) The fabrication of a CS–metal complex. (**B**) Sulfation and calcium compact the conformations of chondroitin [139] (copyright © 2023, American Chemical Society). (**C**) Model for adsorbed complexes showing how proteins anchor hydrated polysaccharides to the surface to enhance lubrication [148] (copyright © 2023, Elsevier).

*CS-metal ion complex*. The modification of CS via metal ions has been the subject of attention for a long time. The most common ions are Na^+^ and K^+^ [131] and other divalent metal ions, including Ca^2+^, Mg^2+^, Mn^2+^, Cu^2+^, Zn^2+^, and Sr^2+^, which can bind to the free carboxyl and sulfate groups instead of the nitrogen atom of *N*-acetyl group [132], forming a kind of polysaccharide–metal complex, which is shown in Figure 6A [4,5,133]. Multiple studies have attributed the binding of CS chains and metal ions to electrostatic interactions, but this is not the only reason [132,134,135]. For instance, the charge density, ionic strength, and calcium/glucuronate ratio can significantly influence the binding of calcium ions and CS [136,137]. Furthermore, calcium ions possess different binding affinities to various glycosaminoglycans. These binding affinities follow a descending order as follows: heparin > CS > keratan sulfate > hyaluronic acid. This order is critically dependent on charge density [137]. Here, dermatan sulfate is not compared. However, another research study indicated that the calcium binding capacity of CS (CS-A) is five times that of dermatan sulfate at a given calcium concentration [138]. Research studies centered around molecular dynamics simulation suggest that calcium ions and sulfation benefit from compacting the conformation of chondroitin (the backbone of CS) in an aqueous solution (Figure 6B) [139]. Instead of sodium ions, calcium ions bind to the carboxyl groups (as opposed to sulfate groups) of CS-A type [140]. This is probably attributable to the preference of Ca^2+^ to bind to carboxyl groups rather than sulfate groups. Interestingly, another research study indicated that CS sulfate groups bind Ca^2+^ more strongly than carboxyl groups [141]. Even so, one thing is for certain: Ca^2+^ can interact with carboxyl and sulfate groups, forming the potential interaction model of −OSO_3_− … Ca^2+^ … −OOC− or −COO− … Ca^2+^ … −OOC− (Figure 6A). Other review papers have noted that previous studies on CS and metal ion binding are mainly concerned with binding kinetics. However, recently, the bioactivity of the chondroitin sulfate metal complex has been the subject of increasing attention. It has been reported that after CS binds to Ca^2+^ or Mg^2+^, its superoxide radical scavenging activity is remarkably improved, and its hydroxyl radical scavenging activity can be enhanced by binding with Ca^2+^, Mg^2+^, Mn^2+^, or Zn^2+^ [133]. In addition, the chondroitin sulfate magnesium complex, chondroitin sulfate strontium complex, and chondroitin sulfate calcium complexes can induce chondrocyte or osteoblast growth for bone care [4,5,6,7]. Here, these complexes are recognized as compounds instead of physical mixtures. In one study, after ion exchange, the calcium holding capacity of CS was more than 4%, while that of strontium was more than 2.7%, and the results of this study indicate that the free carboxyl and sulfate groups of CS chains are able to bind to metal ions.

In addition to metal ions, other components can interact with CS, forming an analogous complex-like conjugate. This can improve the properties of the original substrate. For instance, CS is used in gold nanoparticle synthesis as a reducing or stabilizing agent, forming a CS-capped gold nanoparticle system to deliver insulin via oral administration [142]. CS and methacrylate can be fabricated into nano-capsules via interfacial polymerization to encapsulate the poorly water-soluble drug indomethacin [143]. Zhang and colleagues found that CS-A, together with cisplatin, could form a cisplatin-CS-A conjugate in deionized water and that it was able to reduce the nephrotoxicity induced by cisplatin [144]. Due to its electronegativity and high molecular weight, CS is poorly absorbed by the gastrointestinal tract. Interestingly, Ge and colleagues suggested that using the electrostatic interaction between CS and polyamines (spermine) in aqueous solutions formed a poly-ion conjugate that possessed neutral surface charges, and the absorption efficiency of CS was significantly improved by oral administration [145]. Additionally, the macromolecule is capable of acting with CS to form a conjugate. For instance, chitosan with positive charges can interact with CS to form a polyelectrolyte conjugate comprising a scaffold via spontaneous mixing [146]. This conjugate has blood compatibility, an antibacterial effect, and wound healing properties. Furthermore, recent research suggests that CS is able to interact with the lipid monolayer. Notably, in the presence of Ca^2+^, the head groups together with the head-group-bound water molecules in the dipalmitoyl phosphatidylcholine monolayer, which are significantly impacted by the interaction with CS, indicating that CS displays a linearly chiral secondary structure at a charged biological interface in solution and is likely to form a helical coil [147]. Aside from CS together with other substrates acting as the conjugate status, it can be considered as a mixture with its original status, akin to the mixtures of CS and collagen, CS and glucosamine, and CS and hyaluronic acid, which will be summarized in Section 4.2.

### 3.3. Summary of the Properties of CS

The structural characteristics exhibited by natural CS, including considerable free carboxyl and sulfate groups and its negative charge, make CS interact with several special substrates to change their original properties. CS is known to interact with metal ions, amines, lipid macromolecules, and some other positively charged chemicals to form CS complexes, conjugates, or nanoparticle delivery systems. Interestingly, recent studies have focused on the polysaccharide and protein complexes (or conjugate), such as collagen and gelatin [148,149,150]. Rodrigues and colleagues employed a negatively charged polysaccharide (extracted from *Plantago ovata* seed mucilage) and positively charged lysozyme molecules to form the polysaccharide–protein hydrated complex via electrostatic attraction (Figure 6C), and this complex improved lubrication via stronger adsorption without losing the hydrated thickness of the polyelectrolyte films [148]. The polysaccharide–gelatin interaction occurs due to chemical cross-linking. For example, the fish gelatin–gum arabic complex shell is cross-linked via tannic acid [151]. Additionally, electrostatic interaction, hydrophobic interaction, and hydrogen bond interactions can act as molecular forces, together with external factors such as pH, weight ratio, total biopolymer concentration, and ionic strength [152,153] to stabilize the polysaccharide–protein systems [154,155]. Interesting research on polysaccharide modification via phosphorylation indicates that, due to this process, the apparent structure of polysaccharides changes, and phosphorylated polysaccharides have been shown to exhibit better anti-oxidant activity [156,157,158]. However, few studies regarding CS and protein complexes or phosphorylated CS can be found in the literature. Therefore, it is necessary to investigate the interaction of CS and proteins (e.g., collagen or active peptides), as well as the activity of phosphorylated CS.

## 4. Applications of Chondroitin Sulfate

### 4.1. Functions of Chondroitin Sulfate

*Sulfation-dependent molecular interactions*. The structural characteristics of CS are capable of enhancing the multifaceted biological and pathophysiological functions. The special sulfate groups endow CS with the ability to interact with other molecules showing the functions, as reviewed above in Section 3.2. Research in the literature indicates that the sulfate groups of CS are particularly related to their specific molecular interaction [159,160]. Previous research also suggests that the sulfate group profiles of CS change dramatically, resulting in a 4S/6S ratio increase during brain development [161,162,163], which probably involves the critical period of cortical plasticity. Another exciting study suggested that CS can act as an extracellular gating modifier on voltage-dependent ion channels [164]. Depending on the sulfation position, CS-A and CS-C can bind calcium ions with different affinities and alter voltage-operated ion channel gating by modulating the calcium concentration in the extracellular microenvironment.

**Figure 7:**
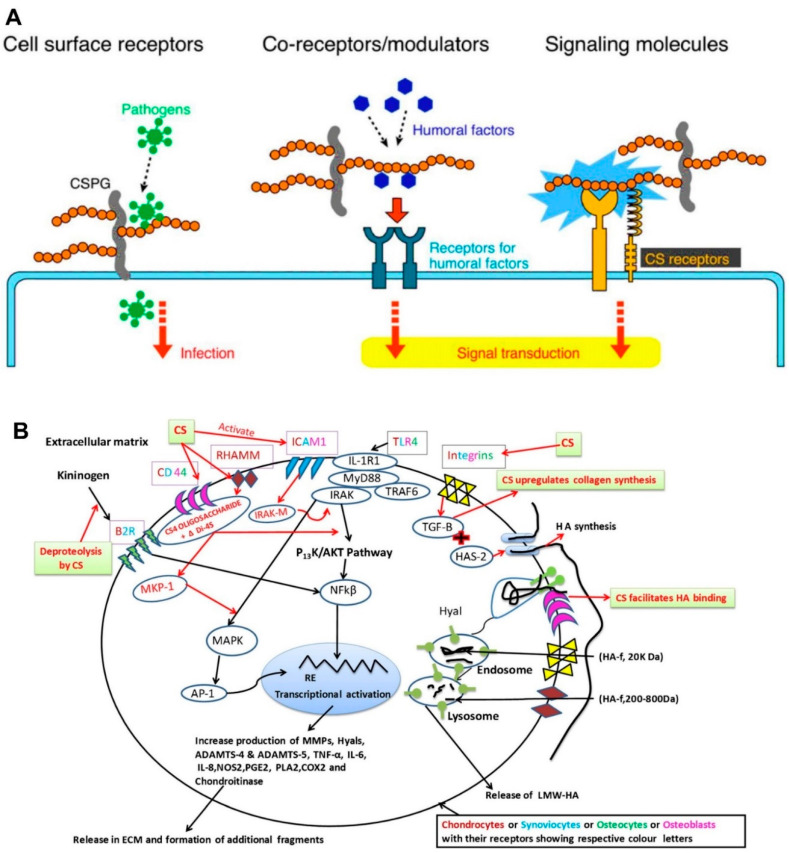
Mechanisms of action regarding CS. (**A**) Receptor or signal modulation of CS chains [1]. (copyright © 2023, Elsevier). (**B**) Anti-inflammatory activity of CS chains. The anti-inflammatory activity of CS with specific inflammatory pathways in different types of cells of osteoarthritis [3]. (copyright © 2023, Springer Science+ Business Media New York). Toll-like receptor-4 (TLR4), myeloid differentiation primary response gene88 (MyD88), interleukin receptor-associated kinase (IRAK), tumor necrosis factor α (TNF-α), TNF receptor-associated factor 6 (TRAF-6), mitogen-activated protein kinase (MAPK), MAPK-phosphatase 1 (MKP-1), response element, specific sequences of DNA (RE), activator protein-1 (AP-1), HA synthase-2 (HAS2), Hyaluronidase (Hyal), aggrecanases (ADAMTS), cyclooxygenase2 (COX-2), interleukin-1 (IL-1), phospholipase A2 (LPA2), matrix metalloproteinases (MMPs), prostaglandin E2 (PGE2), monosulfated disaccharides of CS, sulfated in position 4 (ΔDi-4S).

*As receptors or signal modulators*. Mikami and Kitagawa note that CS is recognized as a cell surface receptor for pathogens, co-receptors and/or signal modulators, and extracellular signaling molecules to exert its functions [1] (Figure 7A). For example, the cell surface CS chains can be a receptor for parasites, bacteria, and viruses to attach and infect the host [165,166,167]. CS, especially for the CS-D or -E units as co-receptors, was demonstrated to bind several humoral factors (e.g., pleiotrophin, midkine, fibroblast growth factor, hepatocyte growth factor, and brain-derived growth factor) to stimulate neurite outgrowth or the proliferation/maintenance of neural stem/progenitor cells [159,160,168,169,170,171]. Chondroitin 4-*O*-sulfotransferase-1, through synthesizing the CS-E-like structure as a signal modulator, is capable of binding the Wnt-3a protein and modulating the β-catenin-dependent Wnt signaling pathway [172,173]. Research in the literature indicates that CS-E mediates contactin-1, inducing the intracellular downstream signaling of contactin-1 and leading to neurite outgrowth; alternatively, prior research also indicates that CS-E binds to N-cadherin, cadherin-11, and bone morphogenetic protein-4, enhancing osteogenic differentiation [174,175,176]. It has been suggested that some CS subtypes can act as an extracellular signaling molecule, further validating the bioactivities of CS.

*Other functions*. Recently, CS was confirmed to possess anti-inflammation activity. It can be a symptomatic slow-acting drug and a structure/disease-modifying anti-osteoarthritis drug employed in osteoarthritis patients [177,178]. CS exhibits anti-inflammation bioactivity directly or indirectly in osteoarthritis patients. Generally, CS, as the biomacromolecule, fails to enter the chondrocytes. It is usually internalized as an oligosaccharide or disaccharide by engaging membrane receptors (e.g., cell surface glycoprotein cluster designation 44 (CD44), a receptor for hyaluronan-mediated motility (RHAMM), and intercellular adhesion molecule1 (ICAM1)) [3] (Figure 7B). Specifically, after engaging CD44 and ICAM1, the interleukin-1 receptor (IL-1R1)-associated kinase-M (IRAK-M) or mitogen-activated protein kinase phosphatase-1 will be released, which could decrease the nuclear translocation of nuclear factor-κB (NF-κB) and the inflammatory reaction. Additionally, the expression of CS-inducing transforming growth factor β1 (TGF-β1) will promote the synthesis of high-molecular-weight hyaluronic acid (HMW-HA) and collagen II. As a result, the binding of extracellular matrix (ECM) fragments or lipopolysaccharides can be affected. Eventually, CS reduces the proteolysis of kininogen to bradykinin (BK), leading to the desensitization and internalization of the bradykinin receptor (B2R) and hindering the signal transduction pathway [179], which simultaneously experiences a decrease in several complement components and inhibits pro-matrix metalloproteinase activation. Additionally, the anti-thrombus, anti-coagulation, anti-oxidative, anti-diabetic, anti-obesity, and other bioactivities of CS have been investigated in vitro and in vivo. It is also believed that CS is beneficial to joint-related pathologies, promoting bone formation and healing and suppressing angiogenesis and tumor growth. In one specific study, CS was also found to regulate blood lipids, cure atherosclerosis, and modulate the repair and regeneration of the central nervous system [10]. Specific information regarding the CS bioactivities mentioned above is listed in Table 2.

### 4.2. Applications of Chondroitin Sulfate

*Medical applications*. CS can be administrated in various forms, including pills, tablets, capsules, powders, or liquids, as well as pharmaceutical-grade injections. Pharmacokinetic studies have shown that CS can be absorbed orally, with bioavailability ranging from 15% to 24%, depending on molecular weight and charge density. The peak plasma level of CS obtained from the tracheal tissue reaches within 1–5 h, while that from the shark source requires 8.7 h [3]. Commercial CS is tolerated, with no significant side effects and drug–drug interactions of overdosages, and it has been confirmed as a safe drug for osteoarthritis treatment by the European League Against Rheumatism Committee [189]. However, one thing should be considered when taking blood thinning medications due to the natural anticoagulant activity of CS. CS is known as the adjuvant therapy for osteoarthritis. The recommended dose for osteoarthritis relief is nearly 1200 mg per day, spread across one or three doses [190]. Moreover, due to its anti-inflammatory activity, the clinical benefits of CS have been identified in symptomatic osteoarthritis of the finger, knee, hip joints, lower back, facial joints, and other diseases [3]. CS and glucosamine are two natural compounds that are used as symptomatic slow-acting drugs against osteoarthritis. In Europe, CS is a registered drug, while it is sold “over the counter” in the form of dietary supplements in North America [191]. As revealed by a database-centered study on the benefits and harms associated with using orally administered chondroitin to treat osteoarthritis compared with a placebo or a comparative oral medication, chondroitin (alone or in combination with glucosamine) is better than the placebo in improving the pain of participants with osteoarthritis in short-term studies, and it has a fewer side effects compared with control [192]. As Bishnoi and colleagues [3] summarized, the reasons why CS has been accepted for use as a symptomatic slow-acting drug are as follows: (1) its ability to slow down osteoarthritis development in clinical trials with significant positive effects [114,193]; (2) its anti-inflammatory activity and beneficial effect on the cartilage or chondrocytes [194]; (3) the fact that its continuous administration can alleviate pain and increase mobility in osteoarthritis patients [195]; (4) its beneficial effect on the formation of new bones, cartilage, and tendons, helping to maintain the structural integrity of tissues and repair damage [86]; (5) the use of antibodies specific for CS epitope in the diagnosis and treatment of connective tissue diseases; (6) its specific biological functions such as cell adhesion, proliferation, morphogenesis, neural network formation, and cell division; and (7) its utilization in veterinary medicine. In addition to treating osteoarthritis, CS can be used in creams [196], eye drops [197,198], and cosmetics [199,200]. It has also been shown to have anti-aging properties [201] and the ability to improve symptoms of bladder inflammation and interstitial cystitis [202,203].

*Biomaterial application*. CS can be employed as the functional component in biomaterials such as functionalized hydrogels, scaffolds, and delivery systems for tissue engineering applications. It has become a key point of focus for researchers because of its favorable biocompatibility, non-toxicity, good biodegradability, and anionic properties, as well as the fact that its use brings no noticeable side effects.

Hydrogels are polymeric networks crosslinked by covalent or physical bonds, and they have the characteristic of being able to absorb large amounts of water. Natural hydrogels based on fibrous proteins and polysaccharides have excellent potential to be used in tissue engineering because of their inherent bioactivity and high cytocompatibility. Hyaluronic acid, another type of GAG, can be functionalized to form hydrogels for tissue engineering [204]. Recently, CS has also been employed in hydrogels for engineering materials. The authors of one study used in tandem CS and hyaluronic acid to form a double-network hydrogel that exhibits improved mechanical strength and structural stability, and this hydrogel could be used as a biomaterial in the form of ink in bioprinting process [205]. Based on the Schiff base reaction between the -NH- functional groups (from N, O-carboxymethyl chitosan) and the -CHO functional groups (from oxidized CS) forming a kind of hydrogel, this hydrogel has injectable, self-healing, antibacterial, and hemostatic properties, enabling its use as a wound dressing material [206]. CS methacrylate-based hydrogels’ applications in cartilage and cardiac regeneration and advanced tissue modeling for tissue engineering have also been investigated [207]. Using CS in concert with other biocompatible polymers can result in the preparation of injectable hydrogels that could be applied in tissue engineering [208,209]. The intention, mechanical, and functional capacity exhibited by a given hydrogel can be enhanced by utilizing a few inorganic ingredients. Due to the unique properties of CS, a methacrylated polyethylene glycol/CS-based hydrogel can bind charged ions of calcium and phosphate and induce effective bone formation with the highest bone mineral density for bone tissue engineering [210]. Another exciting report indicated that CS-based cryogels are biodegradable and hemocompatible, and they can be used safely for tissue engineering, bioreactors, cell separation, or scaffolding materials [211].

The use of CS in the context of scaffolds has been a hot topic in this specific research area in recent years. For instance, CS-containing scaffolds are employed in nucleus pulposus, corneal stromal, tendon, bone, and cartilage tissue engineering [212,213,214,215,216,217,218,219,220]. Moreover, electrospinning nanofibrous scaffolds with CS simulates the skin tissue extracellular matrix to improve cutaneous wound healing [221]. Sadeghi and colleagues fabricated gelatin/polyvinyl alcohol/CS hybrid nanofibrous scaffolds for use in processes such as cell adhesion, cell growth, and cell proliferation without cell toxicity for skin tissue engineering [222]. In cardiac tissue engineering, nanofibrous scaffolds made via gelation and CS contribute to myocardial repair processes [223]. Compared with 2D biomaterials, 3D scaffolds are capable of creating a favorable micro-environment for cell adhesion and growth. For example, CS-modified 3D porous electrospun nanofiber scaffolds can induce cartilage regeneration and modulate inflammation in vivo [224]. Xu and colleagues found that 3D porous chitosan-based CS scaffolds could promote the epithelial-to-mesenchymal transition in prostate cancer cells in vitro [225].

Delivery systems embed drugs, deliver them to a certain destination, and release them under controlled conditions, increasing pharmacodynamic efficacy and reducing the risk of adverse reactions, as well as the toxicity and side effects of the drugs. Currently, nanoparticles [226], hydrogels [227], and microcapsules [228] can be used as carriers in delivery systems. Due to its biocompatibility, biodegradability, non-immunogenicity, and low toxicity, CS has attracted increasing attention for its potential use as a drug delivery system [229]. A common delivery system can be formed via physical crosslinking under the electrostatic interaction of oppositely charged compounds or surfactants. CS, as a typical anionic acid polysaccharide, is able to interact with cationic macromolecules, such as chitosan, to prepare the delivery system [230]. As a type of non-starch polysaccharide, CS is hardly digested by enzymes in the stomach or small intestine, and it is primarily degraded by microorganisms in the colon, which suggests that CS can be an ideal material for colon-targeted drug delivery systems [231]. Additionally, like hyaluronic acid, CS can be a ligand of the CD44 receptor, which is overexpressed in tumor cells [232]. This finding indicates that CS can be employed to synergize tumor-targeted carriers for drug delivery since it has anti-cancer bioactivity [233]. For instance, Liu and colleagues prepared CS-modified doxorubicin nanoparticles with a prominent anti-tumor ability by regulating the CD44 receptor [234]. In addition to drug delivery, gene and cell delivery functions have been associated with the biomaterial applications of CS over the past few years. It has been reported that CS and hyaluronic acid can confer the sorbitan ester-based nanoparticle system with long-term stability and safety for gene delivery in vivo [235]. CS modified on a polyamidoamine dendrimer to form a tumor-targeted carrier for miR-34a delivery can efficiently inhibit tumor growth, indicating that it could be used in tumor gene therapy [236], and CS carries antigens to antigen-presenting cells for cancer immune therapy [237]. Another study also indicated that an injectable CS-type II collagen self-crosslink hydrogel carrier can deliver chondrocytes [238]. Zhou and colleagues used CS hydrogels to deliver adipose-derived stem cells for nucleus pulposus treatment [239]. These results and reports in the literature indicate that CS has great potential to be a safe material (ingredient) with the functions that facilitate drug, gene, and cell delivery in delivery systems.

*Functional food application*. The oral supplementation of CS and glucosamine is recommended by the European Society for Clinical and Economic Aspects of Osteoporosis and Osteoarthritis and Musculoskeletal Diseases and other European Union guidelines for the restoration of the articular cartilage surface in osteoarthritis patients [17]. This means CS, together with glucosamine, has been approved as an active ingredient in dietary supplements for bone care (especially for osteoarthritis). CS is usually used alone or in conjunction with glucosamine for pain relief and to provide anti-inflammatory effects. Although benefits to the osteoarthritic joint tissues have been reported for decades, interestingly, the therapeutic use of glucosamine and CS in clinical settings still appears to be controversial. Recently, an extensive systematic review indicated that neither glucosamine, chondroitin, nor their combination have a significant positive effect on the total Western Ontario and McMaster Universities Osteoarthritis index, and no additional impact was observed using both therapeutic agents in combination for the management of a symptomatic knee [14]. However, according to this analysis, it can be demonstrated that oral supplementation with glucosamine or chondroitin sulfate can reduce pain in knee osteoarthritis. Another interesting study showed that the administration of glucosamine alone decreases bone strength in the femur and fails to reduce the effect on rheumatoid arthritis score in SKG mice. In contrast, these side effects are eliminated when glucosamine is employed in conjunction with chicken cartilage hydrolysates (containing CS and collagen peptides) [240]. Wolff used CS in conjunction with glucosamine to treat the ovariectomized rats for 60 days via gavage, and a two-fold increase in chondrocytes was observed, together with improvements in the remaining cartilage and the trabecular bone compared with the control animals [187]. This means that glucosamine and CS treatment drugs lead to growth plate cell proliferation and bone formation, exhibiting anti-osteoporosis activity. Another study reported that the chondroitin sulfate calcium complex, instead of CS, could increase bone mineral density and alleviate osteoporosis in ovariectomized rats for bone care [241].

In another study, CS was also used as a food supplement for anti-obesity, anti-colon cancer, and modulating gut microbiota. It was administered to rats as a food supplement to a high-fat diet. As indicated by the results, CS could decrease the body weight and parametrial adipose tissue weight and improve fatty liver and hyperlipidemia, which is probably attributed to the inhibition of the small intestinal absorption of dietary fat by suppressing the pancreatic lipase activity and fatty acid uptake via the brush border membrane [242]. Additionally, skate CS oligosaccharides, when to supplement a high-fat diet, exhibit anti-obesity activity by maintaining lower food consumption, inhibiting triglyceride absorption in the intestines, and reducing lipid accumulation [18]. Fucosylated CS (type CS-F) oligosaccharides can regulate lipid disorder in C57BL/6 mice fed a high-fat diet by inhibiting lipid synthesis and facilitating lipidolysis [243]. Moreover, the two characteristics possessed by CS with the recognize-targeted membrane receptor CD44 of the tumor cell and degraded by microorganisms in the colon contribute to this, with great potential as a target anti-colon cancer ingredient in functional foods. Zu and colleagues suggested that CS-functionalized polymeric nanoparticles can load camptothecin for colon cancer-targeted chemotherapy in vitro and in vivo [231]. Wu and colleagues reported that CS from sturgeon could inhibit human colon cell proliferation and induce apoptosis in vitro and in vivo [244]. Another research study suggested that disaccharide CS, when used as a prebiotic, can induce the production of short-chain fatty acids and that it is capable of suppressing human colon cancer cell proliferation and inducing nuclear fragmentation and apoptosis [245]. Recently, there has been increasing evidence of the associations between variations in gut microbiota composition and metabolic disease (e.g., obesity, diabetes, and osteoporosis) [246]. The degradation of CS mainly occurs in the colon, which means an interaction takes place between CS and gut microbiota. Regarding CS fermentation in vitro, *Bacteroides* strains (e.g., *Bacteroides thetaiotaomicron*, *Bacteroides ovatus*, *Bacteroides fragilis*, *Bacteroides stercoris*, *Bacteroides thetaiotaomicron J1*, *Bacteroides thetaiotaomicron 82*, *Bacteroides ovatus E3*, and *Clostridium hathewayi R4*) isolated from human gut microbiota have been demonstrated to degrade CS, and the final degraded product is the disaccharide unit [247]. As revealed by in vivo investigations, the effects exerted by CS and CS oligosaccharides as food supplements on the gut microbiota composition of mice are different, exhibiting a sex-dependent effect [19]. In addition, CS intervention as a food supplement can increase fecal butyrate concentration and ameliorate stress-induced intestinal inflammation [248]. Interestingly, fucosylated CS is capable of reducing the ratio of *Firmicutes* to *Bacteroidetes* by decreasing the abundance of *Lachnospiraceae* and *Allobaculum*. Meanwhile, it can improve the abundance of *Porphyromonadaceae*, *Barnesiella*, and *Bacteroides,* which can alleviate the gut microbiota dysbiosis induced by a high-fat and high-fructose diet [249]. Moreover, dietary fucosylated CS has been shown to exert an anti-inflammation effect by altering gut microbiota in obese mice [250]. The variations in the microbiome or metabolites can affect bone growth and health [251], and animal experiments have also suggested that the gut microbiota is capable of regulating bone mass by modulating immune status, intestinal calcium absorption, and osteoclast-mediated bone resorption [252,253,254]. In another study involving a rat model, the chondroitin sulfate calcium complex was used as a dietary supplement to normal food given to ovariectomized rats and was shown to alleviate their osteoporosis symptoms via gut microbiota alterations [241]. Taken together, CS has the potential to be a functional food due to its bioactivities, which play a partial role in the action of gut microbiota.

### 4.3. Summary of CS Applications

**Figure 8:**
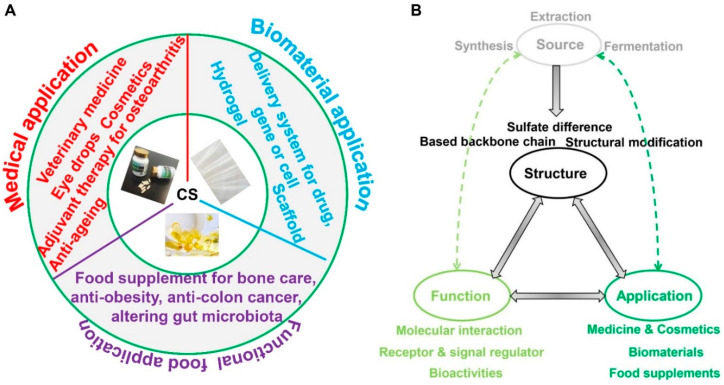
(**A**) The applications of CS. (**B**) The relationships between the sources, structures, functions, and applications of CS.

This section reviews the functions and applications of CS based on the reports in the existing literature (Figure 8A). Although multiple functions of CS have been investigated, most of them are based on cell or animal experimental data, meaning that the clinical utility of CS has rarely been confirmed. Endogenous CS, as a constituent of the extracellular matrix, possesses several functions, such as interacting with receptors or modulating signaling pathways; CS also exhibits bioactivities. Additionally, the use of exogenous intact CS as a macromolecule to contact or enter the tissue cell showing its function is an interesting topic. Moreover, except for its anti-inflammation activity in osteoarthritis treatment, most of the other bioactivities of CS have been studied in cells or rats. Even if oral CS has a therapeutic effect on osteoarthritis, only pain reduction effects have been confirmed. Therefore, the functions or bioactivities of CS in clinical settings require further validation. In fact, clinical data regarding the application of CS, especially in the context of functional foods, are rare. For instance, CS, as a functional component of hydrogels, scaffolds, and delivery systems can be employed in tissue engineering and as a food supplement for obesity, colon cancer, or modulation of gut microbiota, according to the results of numerous animal experiments. Through comparative analyses, the authors of one particularly interesting report suggested that the bioactivities of CS are not equivalent to that of pharmaceuticals and food supplements containing CS [17]. For this report, ten kinds of commercial CS and glucosamine-based food supplements throughout European countries were compared with pharmaceutical CS. Most of the actual CS content in the food supplements was lower than what was stated on the labels, and the CS in the food supplements was of uncertain quality, with equivocal efficacy and doubtful safety in the treatment of osteoarthritis. Hence, the efficacy and safety of CS as a food supplement should be evaluated.

## 5. Conclusions and Future Prospects

A wide variety of sources of CS, including extraction, fermentation, and synthesis, are capable of promoting its diversified structure. CS is endowed with different physicochemical properties for its structural characteristics, which further determines functional diversity. Moreover, its multiple functions facilitate CS applications in a multitude of fields. Accordingly, the target of function and application will guide the various modifications of structure, and the effect of application can further confirm the function, which can be reviewed as the relationship of “Source –Structure –Function –Application” (Figure 8B). Based on this relationship, here, we provide the following perspectives on the prospects of the preparation processes, properties, functions, and applications of CS: (1) Currently, extraction from animal tissue, synthesis using chemical methods, microbial fermentation, and the use of these methods in tandem are the main processes used to prepare CS, and they have been investigated extensively. Regardless of the pharmaceutical application, extraction methods involving pre-treatment in the form of the thermal liquefaction of cartilage are recommended. The eco-friendly co-production of CS and peptides can be achieved using enzyme hydrolysis coupled with membrane separation technology. However, some other purified procedures should be performed under the requirements of higher-quality CS products. Additionally, another method for obtaining CS is bio-synthesis combined with fermentation using genetically engineered strains on a large scale. (2) Structural modifications of CS should be considered, which comprise three aspects. One is to change the molecular weight to investigate oligosaccharide bioactivity. Our understanding of the mechanism of how CS, as a macromolecule, is absorbed and exhibits its activities in vivo is limited. Compared with the intact CS chain, CS in oligosaccharide form is easier to absorb, which may affect its bioactivity. Another method of CS preparation is phosphorylation modification. CS can be derived from sulfidation modifications to chondroitin (the CS backbone). The phosphorylation of polysaccharides has been studied recently. However, research regarding the phosphorylation modification of CS (“chondroitin phosphate”) is scarce. Additionally, the metal ion or bioactive peptide modification of CS is worth considering. (3) Multiple functional activities and applications of CS have been reported in vitro and in vivo; however, studies confirming these functional activities and applications in clinical settings are very limited. Furthermore, the functions of modified CS, including phosphorylation and the application of the combination of CS and other bioactivated compounds such as collagen or peptides, are worth considering. CS, as an active constituent in many applications (e.g., in delivery systems, biomaterials, and functional foods), including in treatments for colon cancer and obesity and modulating gut microbiota, should be the subject of further in depth investigations.

## Figures and Tables

**Table 1:** Results of fermentation processes carried out to prepare CS or CS-like polymers based on wild or engineered bacterial strains.

Strain Source	Process	Product	Yield (g/L)	Ref.
*E. coli*	Batch^+P^	K4 CPS	0.08–0.09	[84]
*E. coli*	Batch^+P^	K4 CPS	0.2	[85]
*E. coli*	Batch	K4 CPS	0.42	[97]
*E. coli*	Batch	K4 CPS	0.3	[98]
*E. coli*	ISPR	K4 CPS	4.7	[87]
*E. coli*	Fed-batch^+P^	Ch	3	[99]
*E. coli*	Batch	K4 CPS	0.41	[100]
*B. subtilis* BN	Batch	CS	4.2	[89]
*B. natto*	Shake flask	CS	0.24	[101]
*E. coli* K4+*kfoC* (*E. coli*)	Batch	K4 CPS+Ch	0.48	[102]
*E. coli* K4 (*mutant kfoC*)	Shake flask	K4 CPS+Ch	0.21	[103]
*E. coli* K4+*rfaH* (*E. coli*)	Fed-batch	K4 CPS+Ch	5.3	[104]
*E. coli* K4+*slyA* (*E. coli*)	Fed-batch	K4 CPS	2.6	[105]
*E. coli* K4+*kfoC* (*E. coli*)	Fed-batch	K4 CPS+Ch	3.5	[106]
*E. coli* K4 (*ΔkfoE*)+*kfoE* (*E. coli*)	Batch	Ch	1.19	[95]
*E. coli* K4 + *pgm, galU, rfaH* (*E. coli*)	Microbioreactor batch	K4 CPS	0.59	[107]
*B. subtilis* + *tuaD* (*B. subtilis*)	Fed-batch	Ch	2.54	[90]
*E. coli* BL21 + *kfoA*, *kfoC*, *kfoF* (*E. coli*)	Fed-batch	Ch	2.4	[108]
*S. equi subsp. Zooepidemicus*+ *kfoA*,*kfoC* (*E. coli*)	Bioreactor batch^+P^	Ch	0.3	[109]
*B. subtilis + tuaD, glmM, kfoA* (*B. subtilis*)	Fed-batch	Ch	7.15	[83]
*E. coli* K4 (*ΔpfkA, mutant kfoC*)*+glmM,glmS,galU,pgm*(*(E. coli*)	DO-stat feeding batch	Fructosylated- Ch	8.43	[110]
*C. glutamicum* (*Δldh*)+ *kfoC*,*kfoA* (*E. coli*)+ *ugdA*(*C. glutamicum*)	Fed-batch	Ch	1.91	[111]
*P. pastoris* + *kfoC*,*kfoA* (*E. coli*)+ *tuaD* (*B. subtilis*)	Fed-batch	Ch	0.19	[11]

Notes: *S. equi subsp. Zooepidemicus*: *Streptococcus equisubsp. Zooepidemicus*; *C. glutamicum*: *Corynebacterium glutamicum*; Batch^+P^: batch fermentation and purification process; ISPR: in situ product removal; K4 CPS: K4 capsular polysaccharide; Ch: chondroitin.

**Table 2:** Specific information regarding CS bioactivities.

Bioactivity	Component	Biological Effects	Ref.
Anti-inflammation	CS	Repress the expression of genes encoding proteolytic enzymes; inhibit IL-1β-induced expression of the pro-inflammatory genes iNOS and COX-2 and restores TGF-β receptors I and II mRNA levels.	[180]
Anti-thrombus	CS-E	Enhances plasminogen activation.	[181]
Anti-coagulation	*O*-sulfonated CS	Increases anti-factor IIa activity and anti-factor Xa activity.	[182]
Anti-oxidation	CS and CS–metal complex	Enhance hydroxyl radical or superoxide radical scavenging activity.	[133]
Anti-tumor	CS-C	Influences tumor-associated inflammation; affects NF-κB signaling and cell behavior and regulates cytokine/chemokine activity.	[183]
Anti-viral	CS-E	Interferes with the binding of viral gC to a CS-E-like receptor on the cell surface.	[167]
Anti-diabetes	CS	Reduces the digestion of carbohydrates; reduces hyperglycemia.	[184]
Anti-obesity	CS	Ameliorates obesity; prevents the gaining of body weight, liver weight, and adipose tissue weight; maintains lower food consumption; inhibits the intestinal absorption of triglyceride; adjusts the serum endotoxin level.	[18]
Neuroprotective	CS sodium salt	Downregulates P-Ser129 α-synuclein and total α-synuclein expression; inhibits ROS overproduction and changes mitochondrion-mediated apoptotic pathways.	[185]
Wound healing	CS aerogel	High hydration and rapid setting to the wound bed.	[186]
Proliferation and bone formation	CS and Glucosamine	Proliferates chondrocytes; increases remaining cartilage and trabecula.	[187]
Protective bladder barrier	CS	Contributes to urothelial barrier function.	[188]

## Data Availability

This study did not report any data.
